# Evaluation of Outcomes Following Surgery for Locally Advanced Pancreatic Neuroendocrine Tumors

**DOI:** 10.1001/jamanetworkopen.2020.24318

**Published:** 2020-11-04

**Authors:** Ashley L. Titan, Jeffrey A. Norton, Andrea T. Fisher, Deshka S. Foster, E. John Harris, David J. Worhunsky, Patrick J. Worth, Monica M. Dua, Brendan C. Visser, George A. Poultsides, Michael T. Longaker, Robert T. Jensen

**Affiliations:** 1 Department of Surgery, Stanford University Hospital, Stanford, California; 2 Department of Surgery, University of Kentucky, Lexington; 3 Gastrointestinal Cell Biology Section, National Institutes of Health, Bethesda, Maryland

## Abstract

**Importance:**

Although outcome of surgical resection of liver metastases from pancreatic neuroendocrine tumors (PNETs) has been extensively studied, little is known about surgery for locally advanced PNETs; it was listed recently by the European neuroendocrine tumor society as a major unmet need.

**Objective:**

To evaluate the outcome of patients who underwent surgery for locally aggressive PNETs.

**Design, Setting, and Participants:**

This retrospective single-center case series reviewed consecutive patients who underwent resection of T3/T4 PNETs at a single academic institution. Data collection occurred from 2003 to 2018. Data analysis was performed in August 2019.

**Main Outcomes and Measures:**

Disease-free survival (primary outcome) and overall mortality (secondary outcome) were assessed with Kaplan-Meier analysis. Recurrence risk (secondary outcome, defined as identification of tumor recurrence on imaging) was assessed with Cox proportional hazard models adjusting for covariates.

**Results:**

In this case series, 99 patients with locally advanced nondistant metastatic PNET (56 men [57%]) with a mean (SEM) age of 57.0 (1.4) years and a mean (SEM) follow-up of 5.3 (0.1) years underwent surgically aggressive resections. Of those, 4 patients (4%) underwent preoperative neoadjuvant treatment (including peptide receptor radionuclide therapy and chemotherapy); 18 patients (18%) underwent pancreaticoduodenectomy, 68 patients (69%) had distal or subtotal pancreatic resection, 10 patients (10%) had total resection, and 3 patients (3%) had other pancreatic procedures. Additional organ resection was required in 86 patients (87%): spleen (71 patients [71%]), major blood vessel (17 patients [17%]), bowel (2 patients [2%]), stomach (4 patients [4%]), and kidney (2 patients [2%]). Five-year disease-free survival was 61% (61 patients) and 5-year overall survival was 91% (91 patients). Of those living, 75 patients (76%) had an Eastern Cooperative Oncology Group score of less than or equal to 1 at last followup. Lymph node involvement (HR, 7.66; 95% CI, 2.78-21.12; *P* < .001), additional organ resected (HR, 6.15; 95% CI, 1.61-23.55; *P* = .008), and male sex (HR, 3.77; 95% CI, 1.68-8.97; *P* = .003) were associated with increased risk of recurrence. Functional tumors had a lower risk of recurrence (HR, 0.23; CI, 0.06-0.89; *P* = .03). Required resection of blood vessels was not associated with a significant increase recurrence risk.

**Conclusions and Relevance:**

In this case series, positive lymph node involvement and resection of organs with tumor involvement were associated with an increased recurrence risk. These subgroups may require adjuvant systemic treatment. These findings suggest that patients with locally advanced PNETs who undergo surgical resection have excellent disease-free and overall survival.

## Key Points

**Question:**

What are the outcomes of patients who undergo aggressive resection of locally advanced pancreatic neuroendocrine tumors (PNETs)?

**Findings:**

In this case series, 99 patients with locally advanced (T3/T4) PNETs and no known distant metastatic disease who underwent resection had a recurrence rate of 35%, but their quality of life remained high, and the overall 5-year survival was 91%.

**Meaning:**

These findings suggest that locally advanced PNETs warrant aggressive surgical resection, including local organs and blood vessels, if necessitated by tumor invasion.

## Teaser

This case series examines data from 2003 to 2018 regarding patients who underwent aggressive resection operative treatment for locally advanced pancreatic neuroendocrine tumors and their outcomes in terms of disease recurrence, quality of life, and overall 5-year survival.

## Introduction

Pancreatic neuroendocrine tumors (PNETs) are a heterogeneous group of epithelial neoplasms that comprise 2% to 7% of all pancreatic tumors.^1,2^ As many as 10% of PNETs may arise in association with a hereditary syndrome, most commonly multiple endocrine neoplasia type 1 (MEN1); however, the majority of PNETs occur sporadically.^3,4^ PNETs can be classified as either functional or nonfunctional depending on whether they secrete a hormone that usually causes a clinical syndrome. Given that most PNETs (60%-90%) are nonfunctional and therefore are not associated with secretion of hormones, patients are unfortunately usually diagnosed with advanced disease for which surgery may not be possible.^5,6^ Complete surgical resection of a PNET has been suggested to be the only potentially curative treatment.^7^ Recently, the European Neuroendocrine Tumor Society identified that assessment of the effects of surgical treatment for locally advanced PNETs is a major unmet need.^1^

This study attempts to address this unmet need by evaluating the outcome of patients who underwent surgery for locally advanced PNETs without distant metastases (including liver metastases). The outcome of surgery for liver metastases from PNETs has been extensively studied previously.^8,9,10,11,12,13,14,15^ However, little is known about surgery for locally advanced PNETs without liver (or other distant) metastases^16^ because it has not, to our knowledge, been previously addressed, likely secondary to the rarity of this presentation. It is important because major surgery to resect locally advanced, possibly nodal metastatic PNETs may be morbid and have life-threatening complications. Further, this aggressive surgery may not improve symptoms and long-term survival compared with newer treatments, such as somatostatin analogues, everolimus, sunitinib, interferon α, and peptide receptor radionuclide therapy (PRRT).^17,18^

We set out to determine the association of resection of locally advanced PNETs with recurrence and mortality using a single academic institution database. We have attempted to define important variables in these patients that are associated with outcomes as defined by recurrence risk, 5-year disease-free survival, and overall survival.

## Methods

This case series study of a single academic institution database of consecutive patients who underwent surgical resection of a locally advanced nondistant metastatic PNET was approved by the Stanford University School of Medicine institutional review board. The requirement for informed patient consent was waived because it was determined that this study did not directly involve human participants.

### Study Population

Patients were included if they had undergone surgical resection of a locally advanced (>4 cm, T3/T4) PNET without distant metastases and with or without lymph node metastases.^19^ Patients were treated between 2003 and 2018. The patients were followed up until death or censored at the cutoff date of November 31, 2019. The records were reviewed by 2 clinicians who were trained to perform standardized medical record abstraction on eligible procedures to supplement the databases. Demographic characteristics, clinical data, and pathological data were retrospectively reviewed and recorded. Age, sex, imaging details, operative findings, tumor characteristics, and postoperative course were recorded. Functionality was assessed according to the presence of a detectable elevated serum level of the relevant hormone associated with a clinical syndrome. Mortality at last follow-up was obtained through a combined review of the medical record, social security index, and the California Cancer Registry. The outcomes measured were overall survival, disease-related survival, disease-free status, time to tumor recurrence, and quality of life as assessed by an Eastern Cooperative Oncology Group (ECOG) score. Recurrence was defined as clear identification of tumor recurrence on computed tomography, magnetic resonance imaging, or gallium 68 DOTATOC scan.

### Statistical Analysis

In the time-to-event analyses, cumulative mortality was estimated by Kaplan-Meier approach and was presented as a curve increasing with time since surgery. Cox proportional hazard models were used to assess risk of recurrence after surgical resection of a locally advanced PNET after adjustment of multiple important demographic characteristics and clinical factors. We performed a stepwise approach for covariate selection and then added additional variables with potential clinical relevance (independent of significance) for inclusion in the model to maximize the risk adjustment. In our stepwise selection process, we used a 2-sided χ^2^ test. All analyses were conducted using Stata version 15 (StataCorp). Statistical significance was defined as *P* < .05, and testing was 2-tailed. Data analysis was performed in August 2019.

## Results

There were 249 patients with PNETs identified. After applying cohort selection criteria, the final study consisted of 99 patients (39.8%) with T3/T4 tumors and no distant metastatic disease (eFigure in the Supplement). Of these 99 patients, the mean (SEM) age was 57 (1.4) years and 56 (57%) were men. Table 1 highlights that the mean (SEM) duration of follow-up was 5.3 (0.06) years and the mean (SEM) follow-up after surgery was 5.1 (0.4) years.

**Table 1.** Patient Demographic Characteristics and Preoperative Tumoral Features as Assessed by Imaging Studies

Characteristic	Patients, No. (%) (N = 99)
Sex	
Men	56 (56.6)
Women	43 (43.4)
Age at surgery, mean (SEM), y	57.0 (1.4)
Previous abdominal surgery	57 (57.6)
Type of PNET	
Nonfunctional	76 (76.8)
Functional	23 (23.2)
Gastrinoma	6 (26.1)
Insulinoma	9 (39.1)
Glucagonoma	3 (13.0)
VIPoma	1 (4.3)
Other	4 (17.4)
Presenting symptoms	
Abdominal pain	18 (18.2)
Diarrhea	7 (7.1)
Gastroesophageal reflux disease	3 (3.0)
Duration of symptoms prior to diagnosis, mean (SEM), y	2.3 (0.7)
MEN1 present	12 (12.1)
Other genetic anomalies	2 (2.0)
Fasting serum gastrin, mean (SEM), pg/mL	636 (110.6)
Pancreatic tumor locations	
Head	21 (21.2)
Neck	4 (4.0)
Body	26 (26.3)
Tail	42 (42.4)
Other	6 (6.1)
Primary tumor size, mean (SEM), cm	4.0 (0.2)
Lymph node involvement	20 (20.2)
Vascular involvement	25 (25.3)
Portal vein	4 (16.0)
Superior mesenteric vein	15 (60.0)
Superior mesenteric artery	3 (12.0)
Other	3 (75.0)
Invasion into surrounding structures	9 (9.1)
Stomach	1 (11.1)
Bowel	2 (22.2)
Spleen	2 (22.2)
Kidney	2 (22.2)
Adrenal	2 (22.2)
Other	1 (11.1)
Preoperative imaging results positive	
CT	88 (88.9)
MRI	25 (25.3)
EUS	35 (35.4)

Abbreviations: CT, computed tomographic; MEN1, multiple endocrine neoplasia type 1; PNET, pancreatic neuroendocrine tumor; MRI, magnetic resonance imaging; VIPoma, vasoactive intestinal peptide tumors; EUS, endoscopic ultrasound.

SI conversion factor: To convert serum gastrin to picomoles per liter, multiply by 0.487.

Of these patients, 12 (12%) had MEN1. Overall, patients presented with the following symptoms: 18 patients (18%) had abdominal pain, 7 patients (7%) had diarrhea, and 3 patients (3%) had gastroesophageal reflux disease. Most locally advanced tumors (76 [77%]) were nonfunctional. Of the functional tumors, 6 (26%) were gastrinomas, 9 (39%) were insulinomas, 3 (13%) were glucagonomas, and 4 (17%) were vasoactive intestinal peptide tumors. All patients with gastrinomas had elevated fasting serum gastrin level (mean, 636 pg/ml; SEM, 100 pg/ml; range, 404-14 000 pg/ml [to convert to picomoles per liter, multiply by 0.481]) (Table 1). Each gastrinoma patient required a proton pump inhibitor to control acid hypersecretion (usual dosage of protonix 80 mg twice daily). All patients with insulinomas were found to have symptomatic hypoglycemia. A minority of patients (4 [4%]) from the overall case series underwent preoperative treatment (including somatostatin analogues and chemotherapy) (Table 2). Patients with severe diarrhea from vasoactive intestinal peptide tumors and necrolytic migratory erythema from glucagonoma had symptoms controlled preoperatively with the somatostatin analogue lanreotide.

**Table 2.** Surgical Findings and Interventions

Characteristic	Patients, No. (%) (N = 99)
Time from presentation to surgery, mean (SEM), y	0.2 (0.1)
Preoperative treatment	
Peptide receptor radionuclide therapy	1 (1.0)
Chemotherapy	3 (3.0)
Primary tumor size, mean (SEM), cm	4 (0.3)
Tumor grade	
1	54 (54.6)
2	30 (30.3)
3	1 (1.0)
Not available	14 (14.1)
Invasion into surrounding structures	
Stomach	4 (4.0)
Bowel	2 (2.0)
Kidney	2 (2.0)
Adrenal	1 (1.0)
Other	1 (1.0)
Lymph node involvement	37 (37.4)
Positive lymph nodes, mean (SEM), No.	3.0 (3.0)
Pancreatic surgical treatment	
Pancreatectomy	
Distal	43 (43.4)
Subtotal	25 (25.3)
Total	10 (10.1)
Whipple resection	18 (18.2)
Enucleation	2 (2.0)
Other	1 (1.0)
Vascular reconstruction	17 (17.2)
Portal vein	9 (9.1)
Superior mesenteric	
Vein	15 (15.2)
Artery	2 (2.0)
Celiac resection	2 (2.0)
Additional resections	
Cholecystectomy	21 (21.2)
Splenectomy	71 (71.7)
Kidney resection	2 (2.0)
Adrenalectomy	1 (1.0)
Bowel resection	2 (2.0)
Gastric resection	4 (4.0)
Positive margins on pathology	16 (16.0)
30-d postoperative mortality	2 (2.0)
Postoperative treatment for advanced disease	16 (16.0)
Somatostatin analogue	13 (13.1)
Chemotherapy	4 (4.0)
Biologics	3 (3.0)
Peptide receptor radionuclide therapy	2 (2.0)
ECOG at last follow up	
0	47 (47.5)
1	28 (28.2)
2	8 (8.0)
3	1 (1.0)
4	0 (0.0)
5	8 (8.0)
Unknown	7 (7.0)

Abbreviation: ECOG, Eastern Cooperative Oncology Group.

**Figure 1.**
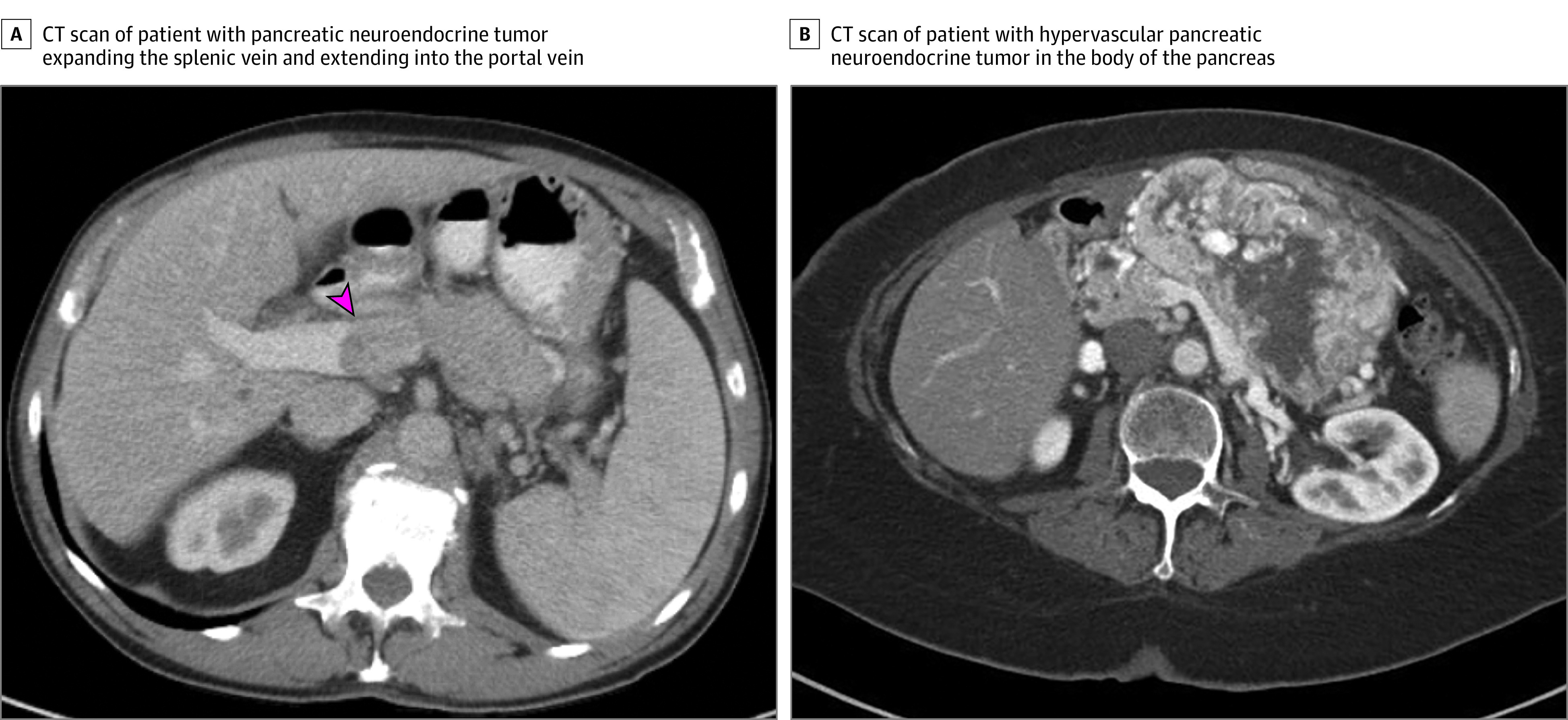
Two Examples of Patients With Locally Advanced Pancreatic Neuroendocrine Tumor Included in This Study A, A computed tomographic (CT) scan showing a patient with an 8 × 2 cm pancreatic neuroendocrine tumor expanding the splenic vein and extending into the portal vein (arrow); B, A CT scan showing another patient with an 8 × 12 cm hypervascular pancreatic neuroendocrine tumor in the body of the pancreas causing mass effect on the splenic vein and porto-splenic confluence.

Each patient underwent advanced imaging studies, most of which were computed tomography scans (88 [89%]) that demonstrated T3/T4 disease without distant metastatic disease (Figure 1 and Table 1). At the time of imaging, the mean (SEM) tumor size was 4 (0.2) cm. The pancreatic tail had the most tumors, which was found in 42 patients (42%). Twenty-five blood vessels demonstrated involvement on imaging. Tumors in 9 patients (9%) had imaging evidence of invasion to surrounding structures (Table 1). There was a mean (SEM) delay of 3 (1.2) months from presentation to surgery. Prior to surgery, 4 patients (4%) underwent neoadjuvant chemotherapy or PRRT to shrink tumor; no additional neoadjuvant treatments were used (ie, biologics or somatostatin receptor antagonists) (Table 2).

After review of the imaging demonstrating a large locally invasive PNET without distant metastases, all patients underwent attempted surgical resection. Most patients (68 [69%]) underwent a distal or subtotal pancreatectomy; 18 patients (18%) underwent pancreaticoduodenectomy, 10 patients (10%) had total pancreatectomy, and 3 patients (3%) had other pancreatic procedures. Concomitant additional organ resection was required in most patients: 71 underwent splenectomy (72%), 21 underwent cholecystectomy (21%), 4 underwent partial gastrectomy (4%), and 2 underwent partial colectomy (2%). Seventeen patients (17%) underwent vascular resection with reconstruction. Most of these required resection of the superior mesenteric vein/portal vein confluence, and 4 patients (4%) had celiac axis or superior mesenteric artery resection (Table 2). Of the vessels identified on preoperative imaging as involved, not all required surgical resection and/or reconstruction; some simply had the tumor dissected off. Thirty-seven patients (37%) of patients who had resections had lymph node involvement with a mean of 3 (range, 0-12) positive lymph nodes. Margins were found to be positive in 16 patients (16%). The 30-day operative mortality was 2 patients (2%). Following surgery, 16 patients (16%) received adjuvant therapy (Table 2).

**Figure 2.**
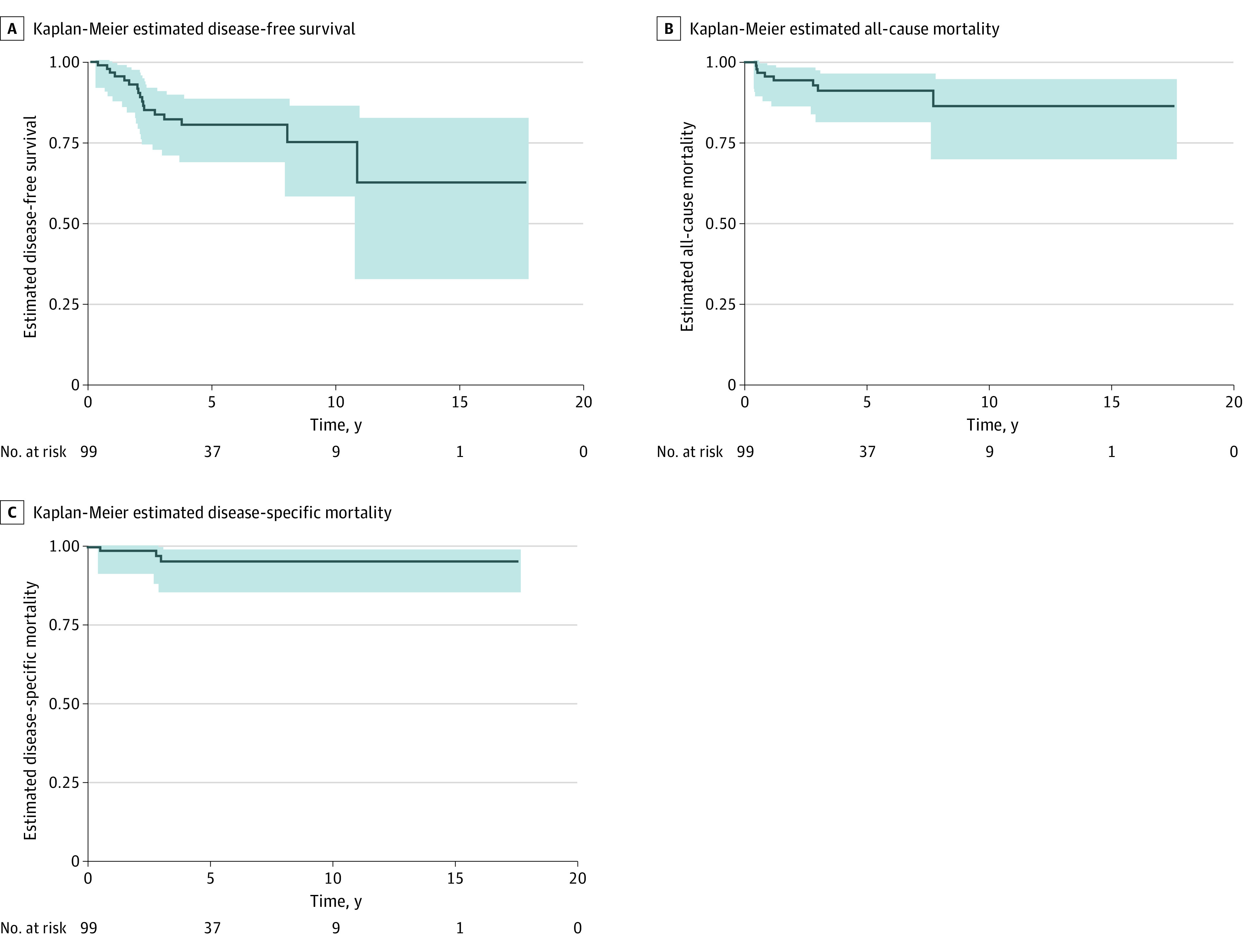
Risk-Adjusted Kaplan-Meier Curves of Patients With Locally Advanced Pancreatic Neuroendocrine Tumor According to Postsurgical Resection Status

At 5 years, the disease-free survival was 61% (61 patients) (Figure 2A), and the overall survival was 91% (91 patients) (Figure 2B). Most deaths (5 patients [62%]) were secondary to disease progression (Figure 2C; eTable 1 in the Supplement). Thirty-five patients (35%) developed recurrent disease; most of which (20 [57%]) were seen in the liver. Of the patients alive at last follow-up, 75 (76%) with or without recurrent tumor had a high quality of life, with an ECOG score of less than or equal to 1 at last follow-up (Table 2). Additionally, 30 patients (30%) were eventually lost to follow-up prior to the end of the study period, however recurrence rate was not significantly different compared with the rest of the cohort.

In our multivariable Cox proportional hazards model, male sex (hazard ratio [HR], 3.77; 95% CI, 1.68-8.97; *P* = .003), lymph node involvement (HR, 7.66; 95% CI, 2.78-21.12; *P* < .001), and additional organ resected (HR, 6.15; 95% CI, 1.61-23.55; *P* = .008) each had a greater probability of tumor recurrence, although functional tumor had a lower risk (HR, 0.23; 95% CI, 0.06-0.89; *P* = .03). The presence of MEN1 (HR, 2.71; 95% CI, 0.90-8.17; *P* = .08), tumor size greater than 6 cm (HR, 0.96; 95% CI, 0.35-2.63; *P* = .93), and tumor location were not associated with an increase in the risk of recurrence (Table 3). Although grade was not found to be significantly associated with recurrence on this multivariable model, there were significantly more patients with G2 disease compared with those with G1 disease (16 patients [53%] vs 14 patients [26%]; *P* = .01) (eTable 2 in the Supplement).

**Table 3.** Cox Proportional Hazards Model of Pancreatic Neuroendocrine Tumor Recurrence Postoperatively

Demographic and clinical factors	HR (95% CI)	*P* value
Age, per y	0.97 (0.94-1.00)	.12
Sex		
Female	1 [Reference]	NA
Male	3.77 (1.68-8.97)	.003
MEN1 positive	2.71 (0.90-8.17)	.08
Tumor >6 cm	0.96 (0.35-2.63)	.93
Functional tumor	0.23 (0.06-0.89)	.03
Grade, per G level	1.04 (0.92-2.01)	.92
Lymph node involvement	7.66 (2.78-21.12)	<.001
Lesion location		
Head	1 [Reference]	NA
Neck	1.05 (0.18-6.17)	.95
Body	0.96 (0.30-3.05)	.95
Tail	0.48 (0.15-1.57)	.22
Multiple or diffuse	1.76 (0.27-1.15)	.07
Other organ resected	6.15 (1.61-23.55)	.008
Vascular resection	1.84 (0.59-5.78)	.30

Abbreviations: HR, hazard ratio; MEN1, multiple endocrine neoplasia type 1; NA, not applicable.

## Discussion

To our knowledge, this study is unique in its evaluation of patients with locally advanced PNETs without distant metastatic disease. Most other publications have focused on either resection of liver or distant metastases to determine whether resection improves survival for those patients^8,9,10,11,12,13,14^ or on the resection of tumors less than 2 cm (T1/T2 tumors),^20^ but this study focused on large, locally advanced tumors. In patients with locally advanced disease, aggressive surgical excision of tumor remains controversial.^1,21,22,23,24^ Most patients in this series were felt to be unresectable by other surgeons and were referred to our center for a second opinion for that reason. A recent study reported that the use of surgery can vary by 4-fold between various centers treating patients with advanced PNETs. This large variation in the use of surgery in these patients was largely because of a lack of information from specific studies in this group of patients and is the primary reason that this area was identified as an important unmet need.^25^

The patient pictured in Figure 1A was diagnosed as unresectable, given the need to resect and reconstruct the portal vein, but he was able to have his tumor completely excised. This study demonstrates that despite previous relative contraindications, there is a role for aggressive surgery in these patients. Patients had an excellent overall survival at 5 years of 91%, with an associated good quality of life, as indicated by low ECOG scores, and an overall recurrence rate of only 35%. Our findings suggest that surgical resection of locally advanced PNETS without distant metastatic disease is indicated.

 Factors associated with tumor recurrence as well as overall survival have not been previously established for locally advanced tumors without metastatic disease. Locally advanced tumors may invade venous structures like the superior mesenteric vein and portal vein, and in these instances they require resection and reconstruction, but in many instances the tumor only abuts the vein without invasion and can be dissected off with blunt and sharp dissection. This was demonstrated by the fact that 25% of patients in our study had preoperative imaging findings suggestive of vascular involvement, but only 17% required resection and reconstruction. Vascular involvement was also not associated with an increased risk of recurrence, further suggesting that vascular resection and reconstruction is warranted. However, there are several retrospective small cohort studies that suggest the contrary.^20,26,27,28,29^ Interestingly, in a study done by US Neuroendocrine Tumor Study Group investigating 873 patients with nonfunctional localized (no distant metastatic disease) PNET that underwent surgical resection, recurrence-free survival was found to be associated with a multitude of factors, including tumor burden and lymph node involvement but not vascular involvement.^26^ The 5-year disease-free survival in patients found to have locally advanced tumor was 60%, which was similar to our findings.^26^ However, the current study demonstrated that male sex, lymph node metastases, and resection of additional organs (but not blood vessels) were associated with an increased probability of tumor recurrence. Interestingly, functional tumors had a lower incidence of recurrence. Our findings support prior studies that have found that men are at a greater risk of disease recurrence postoperatively.^30,31^ Additionally, men have been shown to have a greater incidence of PNETs compared with women, suggesting that there are underlying sex differences that contribute to the pathogenesis of these neoplasms^32^; however, exact factors responsible for such differences have yet to be fully elucidated.

Since the beginning of this study in 2003, new options for medical management of these patients have been developed, with the most recent being PRRT.^33^ In addition to PRRT, recent phase 3 trials have led to the approval of everolimus,^34^ sunitinib,^35^ and somatostatin analogues^36^ to treat patients with advanced PNETs. Each of these treatments has been shown to prolong progression-free survival. Although they have not been shown to extend overall survival, each are now being increasingly used in patients with advanced PNETs. Among others,^37,38^ these previous studies indicate that upfront therapy with either PRRT or the chemotherapy listed does reduce tumor burden and therefore may improve the ability to perform a successful resection on patients with locally advanced PNET. Prior to the availability of these newer nonsurgical treatments, there were few treatment alternatives.

The recent approval of these other nonsurgical treatments has increased the relevance of this study, which defines the beneficial role of surgery in patients with locally advanced PNETs. It provides the information necessary for the clinician to better select an appropriate antitumor treatment.

### Limitations

This study has several limitations. Given that all patients included in the present study underwent surgery, there is no control group of similar patients who did not undergo surgery. There is also a selection bias toward operative treatment. We elected to include only patients who underwent surgery because having complete pathological analysis of the extent of tumor makes the survival data more meaningful. Additionally, tumor grade was not available for 14 patients, which may have skewed our multivariable model’s finding regarding the lack of association of grade with recurrence risk, which has been well-established in the literature and was observed with our univariate analysis of patients with G2 tumors compared with patients with G1 tumors.

## Conclusions

This study’s findings suggest that patients with locally advanced PNETs without liver or distant metastases can have the tumor excised with acceptable rates of disease-free progression and mortality. Furthermore, 61 patients (61%) remained disease-free at 5 years postsurgery, and 91 patients (91%) were alive with excellent quality of life. These suggest that aggressive surgical resection can provide excellent outcomes for these patients.

## Notes

Supplement. **eFigure.** Flow Diagram Outlining Cohort Selection for the Current Study **eTable 1.** Recurrence and Mortality **eTable 2.** Recurrence Based on Tumor Grade
