# Supplementary material for: Evaluation of Outcomes Following Surgery for Locally Advanced Pancreatic Neuroendocrine Tumors
Source: JAMA Netw Open. 2020 Nov 4;3(11):e2024318. doi: 10.1001/jamanetworkopen.2020.24318 (PMC7643030; doi:10.1001/jamanetworkopen.2020.24318)
Supplement: Supplement. — eFigure. Flow Diagram Outlining Cohort Selection for the Current Study eTable 1. Recurrence and Mortality eTable 2. Recurrence Based on Tumor Grade [file jamanetwopen-e2024318-s001.pdf]

Supplemental Online Content

Titan AL, Norton JA, Fisher AT, et al. Evaluation of outcomes following surgery for locally advanced pancreatic neuroendocrine tumors. *JAMA Netw Open*. 2020;3(11):e2024318. doi:10.1001/jamanetworkopen.2020.24318

eFigure. Flow Diagram Outlining Cohort Selection for the Current Study
eTable 1. Recurrence and Mortality
eTable 2. Recurrence Based on Tumor Grade

This supplemental material has been provided by the authors to give readers additional information about their work.

© 2020 Titan et al. *JAMA Network Open.*

Supplemental Figure 1:

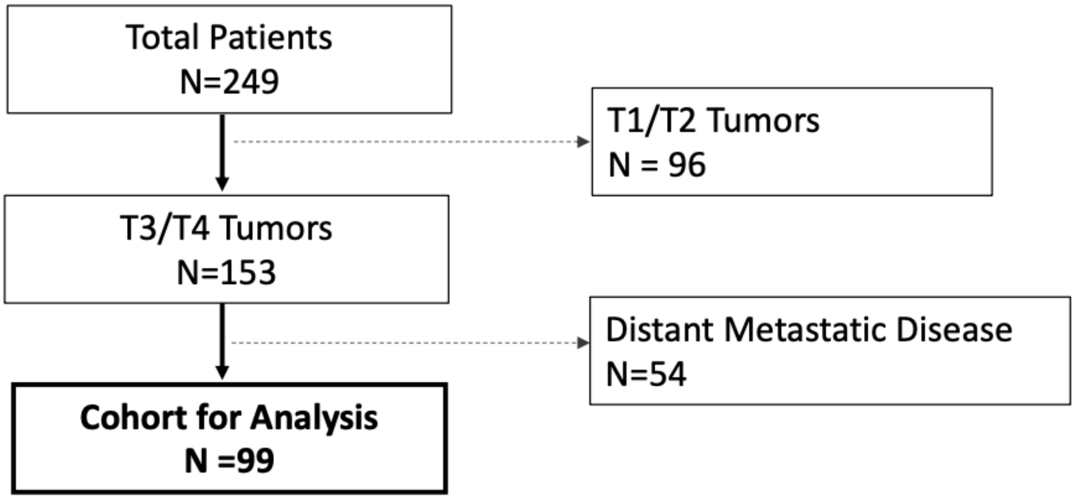

© 2020 Titan et al. *JAMA Network Open.*

Supplemental Table 1: Recurrence and Mortality

Outcomes	Total Population	
**Duration of follow-up after diagnosis, y (SEM)**	5.3	(0.06)
Range, y	0.04-17.1	
**Duration of follow up after surgery, y (SEM)**	5.1	(0.4)
Range, y	0.02-17.7	
**Result of surgery**		
**Alive last Follow up, n (%)**	91	(91.9)
Alive and disease-free	59	(59.6)
Alive with disease	32	(32.3)
**Recurrent Disease, n (%)**	35	(35.4)
Time to recurrence, y (SEM)	4.4	(0.4)
Range, y	0.04-17.6	
**Location of Recurrence, n (%)**		
Liver	17	(48.6)
Pancreas/Pancreatic Bed	8	(22.9)
Common hepatic duct	1	(2.9)
Omental and abdominal wall	1	(2.9)
Gastric	1	(2.9)
Local lymph node	5	(14.2)
Liver and sites of recurrence	3	(8.6)
**Dead last follow up, n (%)**	8	(8.1)
Disease-specific mortality	3	(3.0)
Non-disease-specific mortality	5	(5.1)
**Time from surgery to death, y (SEM)**	3.3	(0.9)
Range, y	0.5-11.8	

© 2020 Titan et al. *JAMA Network Open.*

Supplemental Table 2: Recurrence based on tumor grade

	G1		G2		G3		Not Available	
**Recurrence, n (%)**	54	(100.0)	30	(100.0)	1	(100.0)	14	(100.0)
No	40	(74.1)	14	(46.7)	1	100	9	(57.1)
Yes	14	(25.9)	16	(53.3)	0	0	5	(35.7)

© 2020 Titan et al. *JAMA Network Open.*
